# Pediatric Emergency Medicine Simulation Curriculum: Submersion Injury With Hypothermia and Ventricular Fibrillation

**DOI:** 10.15766/mep_2374-8265.10643

**Published:** 2017-10-17

**Authors:** Anita Thomas, Elizabeth Sanseau, Neil Uspal, Rebekah Burns, Marc Auerbach, Derya Caglar, Kimberly Stone, Jennifer Reid

**Affiliations:** 1 Assistant Professor, Department of Pediatrics, Division of Emergency Medicine, University of Washington School of Medicine; 2 Assistant Professor, Department of Pediatrics, Division of Emergency Medicine, Seattle Children's Hospital; 3 Pediatric Resident, Seattle Children's Hospital; 4 Pediatric Resident, University of Washington School of Medicine; 5 Associate Professor of Pediatric Emergency Medicine and Emergency Medicine, Department of Pediatrics, Yale School of Medicine; 6 Associate Professor of Pediatric Emergency Medicine and Emergency Medicine, Department of Pediatrics, Yale-New Haven Children's Hospital; 7 Associate Professor, Department of Pediatrics, Division of Emergency Medicine, University of Washington School of Medicine; 8 Associate Professor, Department of Pediatrics, Division of Emergency Medicine, Seattle Children's Hospital; 9 Co-Director of Pediatric Emergency Medicine Simulation, Seattle Children's Hospital

**Keywords:** Simulation, Ventricular Fibrillation, Hypothermia, Drowning, Pediatric Emergency Medicine, Submersion, Immersion

## Abstract

**Introduction:**

Submersion injury or drowning is a leading preventable cause of pediatric mortality and morbidity. Submersion injuries are often accompanied by hypothermia and asphyxia that can lead to inadequate oxygen delivery to tissues and subsequent cardiac arrhythmias.

**Methods:**

This simulation-based curriculum involves the identification and management of a submersion injury in a 4-year-old boy who was rescued from a cold-water submersion. The simulated patient is apneic, pulseless, bradycardic, and hypothermic; he is being bag-mask ventilated on arrival without intravenous access. He ultimately develops ventricular fibrillation. Providers must recognize the degree of submersion injury, initiate early airway protection, adequately address circulation, and be alert to developing hypothermia and cardiac arrhythmias to prevent further decompensation. This scenario can be modified based on trainee level (pediatric residents vs. pediatric emergency medicine fellows).

**Results:**

A total of 22 trainees (PGY 1-PGY 6 pediatric residents and pediatric emergency medicine fellows) participated in this simulation curriculum on separate occasions and rated it as an overall positive learning experience. The curriculum's goal is to provide learners with an opportunity to manage life-threatening pediatric submersion injuries, where the correct steps need to be taken in a limited period of time.

**Discussion:**

We have provided preparatory materials to help instructors set up, run, and debrief the scenario in a standardized fashion. The debriefing tools allow for adaptation depending on learners' needs and individual experiences during the simulated scenario. Also included are supporting educational materials and a learner feedback form that can be used to evaluate the session.

## Educational Objectives

By the end of the session, learners will be able to:

1. Perform a primary survey of a pediatric patient with a submersion injury.
2. Elicit critical history (e.g., length of submersion, mechanism of submersion, water temperature, prehospital vital signs/treatment/observation).
3. Recognize the need to call for team assistance early in an event (e.g., calling a rapid response or code blue), especially if there are not many team members initially in the room evaluating the patient.
4. Demonstrate effective teamwork and communication skills.
5. Recognize the degree and progression of submersion injuries (aspiration, laryngospasm, acute respiratory distress syndrome, hypothermia, cardiac arrhythmias) and demonstrate how to appropriately treat submersion injuries.
6. Understand prognosis and prevention of submersion injuries in order to communicate with families.

## Introduction

The purpose of this curriculum is simulation education in management of submersion injuries or drowning. Effective care of this potentially life-threatening medical emergency requires the ability to stabilize the patient while simultaneously identifying the pertinent risk factors and anticipating possible pathophysiology sequelae.

Drowning is one of the leading causes of childhood morbidity and mortality in the world.^1^ The World Congress of Drowning defines drowning as “the process of experiencing respiratory impairment from submersion/immersion in liquid.”^2^ Drowning can be fatal or nonfatal, but when compared with other types of injuries, drowning has one of the highest fatality rates. Prevention is the most important step to reduce the impact of drowning injury, followed by early initiation of cardiopulmonary resuscitation (CPR) at the scene. Death rates due to fatal drowning follow a bimodal pattern, with spikes at ages 1–4 years and 15–19 years. Risk factors for drowning include male sex, use of alcohol or drugs, low income, poor education, rural residency, aquatic exposure, risky behavior, lack of supervision, and medical conditions such as epilepsy and arrhythmias.^3^ Submersion pathophysiology may result in water aspiration and laryngospasm, leading to apnea, loss of consciousness, and resultant brain hypoxia.^4,5^

The goals of management are to reverse anoxia from submersion, improve cerebral perfusion, and limit secondary hypoxic injury, hypothermia, and dysrhythmias. This can be accomplished by ensuring an adequate airway as well as adequate breathing and circulation. Cardiac arrest from drowning is due to hypoxia; therefore, CPR should follow the traditional ABC (airway-breathing-circulation) sequence rather than the CAB (circulation-airway-breathing) sequence with CPR initiated and defibrillator application indicated by Pediatric Advanced Life Support (PALS).^4,6^ Fluids and vasopressor medications are often required in patients with submersion injury, and expedient vascular or intraosseous access should be established as quickly as possible. Recognition and treatment of hypothermia are key, as cases of moderate to severe hypothermia can depress myocardial function and cause arrhythmias.^4^ Thus, prevention and treatment are pivotal via removing damp clothing and initiating rewarming.^4,7^

Adult learning principles were considered during the design of this curriculum. The simulation allows for active learning through participation and is designed to reinforce illness scripts. Because it requires participants to function as a team, it also offers the opportunity to practice and refine team communication, leadership, and followership skills. This simulation curriculum allows learners to perform a systematic primary survey, discuss the history and prognostic factors of the submersion patient, and demonstrate the treatment of hypothermia and cardiac arrhythmias secondary to submersion. This curriculum may be used independently or in conjunction with other simulation-based curricula from the Pediatric Emergency Medicine Simulation Curriculum available on *MedEdPORTAL.*^8–20^

This curriculum was originally developed for a target audience of pediatric emergency medicine fellows. It would also be appropriate for pediatric and emergency medicine residents, nurses, and respiratory therapists. As pediatric submersion injuries are high-risk, low-frequency events, it is ideal to practice this scenario in an interprofessional fashion to ensure learning and appropriate teamwork across all levels of medical providers. Ideally, participants should have prerequisite knowledge of how to perform a primary and secondary survey as well as manage emergent issues with the airway, lungs, or circulatory system. Preparticipation review of these skills may be helpful for some learners.^21–23^

## Methods

This simulation case was designed to help learners systematically recognize and manage submersion injuries in addition to focusing on teamwork and communication skills. Specifically, through participation in the simulation, the learners demonstrate how to perform an initial patient assessment, apply appropriate monitoring, and identify the submersion injury. The learners demonstrate appropriate management of airway and breathing with supplemental oxygen and a secured airway, management of hypothermia, management of arrhythmias, proper equipment setup, and technical skills. They coordinate a proper team structure, assign leadership, and demonstrate effective communication skills. The simulation scenario (Appendix A); environment preparation (Appendix B); chest X-ray, electrocardiogram, and rhythm strip (Appendix C); teamwork and communication (TeamSTEPPS) glossary (Appendix D—we recommend that participants review the TeamSTEPPS website if unfamiliar with this safety teamwork process)^24^; debriefing materials (Appendix E); evaluation form (Appendix F); and PowerPoint presentation (Appendix G) are provided for the instructor in preparation for the simulation case.

### Equipment/Environment

The setting is an emergency department (ED) resuscitation room, and the simulation can be conducted in an ED resuscitation room or in a simulation lab. We used a high-fidelity child mannequin. See the environment preparation document (Appendix B) for a complete list of suggested equipment. The patient arrives on a stretcher via ambulance driven by emergency medical personnel. He is wearing wet or damp clothes. Active CPR is being performed, and there is no IV access or endotracheal intubation. The team is told that the patient is a 4-year-old boy who presents after being submerged in water for 10 minutes after he fell from a dock. Printouts or electronic versions of the chest X-ray, electrocardiogram, and rhythm strip (Appendix C) are made available to trainees upon request. The simulation instructor provides verbal clinical changes and laboratory findings throughout the scenario.

If using a low-fidelity mannequin, vital signs may be provided verbally or via a simulator application for a phone or tablet. Physical exam findings can be described concurrently with the learners' examination of the mannequin.

### Personnel

This simulation scenario can be adjusted to accommodate three to seven trainees per session, with a target audience of pediatric emergency medicine health care providers, including pediatric, family medicine, and emergency medicine residents, fellows, attending physicians, respiratory therapists, and nurses. There are no prerequisites for trainees prior to participating in the case. Depending on the trainees' backgrounds, additional learning tools may be provided to the learners prior to participating in the simulation. See the reference list for recommended background preparation materials.^4,21,24,25^ Realism and maximal learning are attained if all participants are functioning in their normal roles (i.e., nurses perform nursing roles, physician perform physician roles) and with the number of participants expected in the health care team normally. For example, if a more experienced nurse would function in the role of bedside nurse, than he or she should play that role, although this may be adjusted depending on learner needs. In order to assure appropriate teamwork and communication among various levels of medical providers, it is ideal and most realistic if this simulation is performed in a multidisciplinary fashion. Trainees or confederates can act in unfilled roles or these roles may be left unfilled, although this may compromise realism and make some learning objectives more difficult to achieve.

### Assessment and Debriefing

After completion of the simulation scenario, twice the amount of time should be allowed for debriefing with trainees and facilitators. The debriefing session is a valuable component of simulation education, and care should be taken to allow adequate time for it. The debriefing materials (Appendix E) can be used to facilitate the debriefing discussion and provide formative feedback (assessment) with regard to medical management and teamwork/communication. The simulation session evaluation form (Appendix F) is used to obtain feedback and evaluation on the simulation session from the participants. The PowerPoint slides (Appendix G) are optional and can be used to help deliver supplemental content knowledge regarding submersion injuries in children and the diagnosis and management of this problem. We chose to utilize the supplemental PowerPoint slides after the simulation, but they may be used prior to the simulation depending on learner needs.

## Results

We have implemented this curriculum with three pediatric emergency fellows and 19 pediatric residents at various stages of their training at our institution. The curriculum received positive feedback from the participants. All 22 participants strongly agreed (5 on our 5-point Likert scale) with the following statements: “The simulation case provided is relevant to my work,” “This simulation case was effective in teaching basic resuscitation skills,” “This simulation case was effective in teaching submersion injury management skills,” “The debrief created a safe environment,” and “The debrief promoted reflection and team discussion.” Twenty participants strongly agreed (5 on our 5-point Likert scale) with the statement “The simulation case was realistic,” and two participants rated that statement 4 on our 5-point Likert scale, or agreed. Participants reported that their clinical practice has changed due to the knowledge and skills obtained through this curriculum. Specifically, participants reported that they think more about trauma with drowning and recognize the importance of ventilation with the submersion patient. Participants reported a decreased sense of realism when the session was performed in the simulation lab. Some specific examples of participant responses to prompts appear below.

Can you list/describe one or more ways this simulation session will change how you do your job?

• “Great to review management of submersion injury, especially <10 vs. >10 minute prognostics.”
• “Great refresher on PALS, chest rise with bagging, hypothermia, and drowning.”
• “Emphasis on importance of ventilation.”
• “Continue resuscitation until warmed. Initiate warming early! Speak calmly, loudly, and directly to people.”
• “Thinking about trauma with drowning.”
• “Management of submersion injuries.”
• “Remember ways to warm with hypothermia.”
• “Get pads on right away.”
• “Look at PALS algorithms.”
• “Feels chaotic—summarizing helps.”
• “Practice prioritizing multiple info and leading a code.”

How could we improve this scenario?

• “On debrief review all jobs that people did. I think the survey doc wanted a little more feedback.”
• “The location was a bit tough (sim center vs. resuscitation room). Team did great job adjusting.”

General comments included the following:

• “Great at clearly stating and working towards learning objectives.”
• “I thought it was one of the best mock codes—perfect amount of chaos that forced loud and clear communication.”
• “Great debriefing session highlighting the strengths of the team.”
• “Excellent debrief with good succinct learning points.”

## Discussion

This resource is designed as a comprehensive curriculum to support instructors in teaching the recognition and management of submersion injury in a pediatric patient to a wide variety of learner groups. This case is a low-frequency, high-risk scenario that pediatric emergency medicine providers must be familiar with in order to provide expedient and appropriate care.

Trialing this scenario was crucial to refining the curriculum material. We found it useful to adjust the scenario based on the level of learner training. Specifically, with the pediatric emergency fellows, we required two rounds of IV epinephrine and two rounds of defibrillation to stabilize the patient. For the pediatric resident learners, we modified the scenario to include only one round of IV epinephrine and one round of defibrillation for stabilization. This adjustment was made to fit time constraints for resident trainees as well as to allow for fewer interventions to restore a perfusing cardiac rhythm since resident learners have less training than fellow learners. Moving forward, we would like to include nursing staff in this scenario in all roles—as confederates, learners, and facilitators. At our institution, learners and facilitators often have to role-play as nurses, which can diminish realism. A limitation noted during an implementation with pediatric emergency medicine fellows was that the simulation had to be conducted in the simulation center (vs. in our ED resuscitation room), which made it difficult for one learner to suspend disbelief. In situ simulation is ideal, albeit not feasible at all times, due to workflow and staffing constraints. Thus, we would also prefer to conduct this scenario in situ in an effort to optimize the realism and therefore the effect of the simulation. In addition, our evaluation approach is primarily perception centered and does not measure specific objectives. We attempted to revise our learner evaluation to better reflect our learning objectives (evaluation questions 4–10), although this did increase the number of evaluation questions. This scenario will continue to be used as part of the recurring pediatric emergency medicine fellow and pediatric resident simulation curriculum at our institution.

We have attempted to limit resources needed to run this simulation scenario and execute its curricular goals. There are cues within the scenario that allow the instructor to respond to learners while the scenario is ongoing. The debriefing materials have been created to address potential issues during the scenario as well as to allow instructors to modify their educational objectives to fit their targeted audience.

## Appendices

A. Simulation Case.docx
B. Environment Preparation.docx
C. CXR ECG Rhythm Strip.docx
D. Teamwork and Communication Glossary.docx
E. Debriefing Materials.docx
F. Session Evaluation Form.docx
G. PowerPoint Presentation.ppt All appendices are peer reviewed as integral parts of the Original Publication.
