# Supplementary material for: Pediatric Emergency Medicine Simulation Curriculum: Submersion Injury With Hypothermia and Ventricular Fibrillation
Source: MedEdPORTAL. 2017 Oct 17;13:10643. doi: 10.15766/mep_2374-8265.10643 (PMC6338133; doi:10.15766/mep_2374-8265.10643)
Supplement: Supplementary file 1 — A. Simulation Case.docx B. Environment Preparation.docx C. CXR ECG Rhythm Strip.docx D. Teamwork and Communication Glossary.docx E. Debriefing Materials.docx F. Session Evaluation Form.docx G. PowerPoint Presentation.ppt [file mep-13-10643-s001.zip › A. Simulation Case.docx]

| **Appendix A: MedEdPORTAL Simulation Case**  **SIMULATION CASE TITLE:** Pediatric Emergency Medicine Simulation Curriculum: Submersion Injury with Hypothermia and Ventricular Fibrillation  AUTHORS: Thomas Anita, MD, Sanseau Elizabeth, MD, Uspal Neil, MD, Burns Rebekah, MD, Auerbach Marc, MD, Caglar Derya, MD, Stone Kimberly, MD, and Reid Jennifer, MD, | |
| --- | --- |
| **PATIENT NAME:** Jack  **PATIENT AGE:** 4 years old  **PATIENT WEIGHT:** 15 kg  **CHIEF COMPLAINT:** Submersion injury | |
|  | |
| **Brief narrative description of case** | Jack was seen stumbling off of a dock, falling in between the dock and a boat. He was rescued by boaters, approximately 10 minutes after seeing him fall in the water. Cardiopulmonary resuscitation (CPR) was initiated at the scene, and EMS arrived 11 minutes later. He was apneic and pulseless when they arrived. Unable to obtain (UTO) endotracheal tube in field – receiving bag-mask ventilation by medics on arrival with baseline heart rate (HR) of 40 and active CPR in progress. No intravenous (IV) or intraosseous (IO) access. The patient is in wet clothes and on cardiac monitors. Initially, the patient is bradycardic and hypothermic.  Anticipated interventions include primary assessment, continuation of bradycardia management, and initiation of hypothermia and submersion management, as well as establishing vascular access. From initial rhythm of bradycardia, the patient subsequently develops ventricular fibrillation. Anticipated interventions include supporting Airway, Breathing, Circulation (ABCs), endotracheal intubation, and Pediatric Advanced Life Support management of ventricular fibrillation. The patient stabilizes after the first defibrillation (2J/kg) when run with residents, after the second defibrillation (4J/kg) when run with fellows. |
| **Primary Learning Objectives** | - Recognize the signs and symptoms of drowning - Manage submersion injuries - Manage bradycardia with poor perfusion - Manage ventricular fibrillation - Crisis resource management |
| **Critical Actions** | - Secure airway - Obtain IV/IO access - Manage dysrhythmias - Manage hypothermia - Manage potential trauma - Recognize potential ingestions (alcohol (ETOH)/drugs) - Division of labor to accomplish multiple tasks simultaneously - Discussion amongst participants about likely diagnosis and appropriate management - Clear communication with team members - Open communication with the parent even if there is uncertainty |
| **Learner Preparation** | - Introduction to the concept of specific team roles in resuscitation   - Farah MM, Tay K-Y, Lavelle J. A general approach to ill and injured children. In: Shaw KN, Bachur RG, eds. Fleisher & Ludwig’s Textbook of Pediatric Emergency Medicine. 7th ed. Philadelphia, PA: Wolters Kluwer; 2016:1-8. - General knowledge about submersion injuries   - Szpilman, David, et al. "Drowning." *New England journal of medicine* 366.22 (2012): 2102-2110.   - Quan, Linda, Christopher D. Mack, and Melissa A. Schiff. "Association of water temperature and submersion duration and drowning outcome." *Resuscitation* 85.6 (2014): 790-794. |

| Initial Presentation | | | |
| --- | --- | --- | --- |
| **Initial vital signs** | Heart rate (HR) 34 Oxygen saturation (SpO2) 85% Blood Pressure (BP) Unable to Obtain (UTO) Respiratory Rate (RR) bag mask ventilated Temperature (T) 32 degrees Celsius | | |
| Overall Appearance | Jack was seen stumbling off of a dock, falling in between the dock and a boat. He was rescued by boaters, approximately 10 minutes after seeing him fall in the water. CPR was initiated at the scene, and EMS arrived 11 minutes later. He was apneic and pulseless when they arrived, now with HR 40. Unable to obtain endotracheal tube in field – bagging on arrival with active CPR in progress. Unable to obtain IV or IO access in the field so were unable to give epinephrine. The patient is in wet clothes and on cardiac monitors. Initially, the patient is bradycardic and hypothermic.  The patient is brought in by EMS and a parent. There are 3 medics: one pushing the gurney, one bagging the patient and one doing CPR.  ( __ ) will play Medic 1  ( __ ) will play Medic 2  ( __ ) will play Medic 3  ( __ ) will play the Parent  *Of note, these roles can be played by confederates such as the simulation center team. If confederates are limited, recommend starting the scenario in the room with the patient already arrived with 2 instead of 3 medics (one bagging the patient and one doing CPR) | | |
| **Actors and roles in the room at case start** | Doctor #1: Team Leader  Doctor #2: Airway Physician (MD)  Doctor #3: Survey MD  Nurse #1: Medication Administration Nurse (RN)  Nurse #2: Medication Preparation RN (optional)  Nurse #3: Documenting RN (optional)  Nurse #4: Circulating RN (optional)  Instructor #1: Simulation instructor who will also act as debriefer  Instructor #2: If a 2^nd^ instructor is available, cast them as “parent,” available to answer questions and assist with debrief. If a 2^nd^ instructor is not available, the facilitator can play the role of mom as well. | | |
| **HPI** | Jack is a 4-year-old previously healthy male with no medical problems seen stumbling off of a dock, falling in between the dock and a boat, and was noted to be rescued about 10 minutes after onset of submersion.  When asked about events leading up to the event (SAMPLE):  SAMPLE history:  Signs/symptoms (sx)- found cyanotic, apneic, pulseless after ~10 minutes of water submersion (see above)  Allergies- none  Medications- none  Past Medical History: Full term, no complications, immunizations up to date. No hospitalizations/surgeries.  Last meal at 7am  Events preceding- Patient wandering on dock with parents when he tripped and fell between the dock and a boat and rescued about 10 minutes after onset of submersion  If asked for review of systems:  Negative Review of Systems (ROS) ROS prior to injury  If asked about home environment/social history:  Lives with Mother and Father. Attends pre-school. No other caregivers. Mom has no concern of non-accidental trauma. No known sick contacts. | | |
| **Past Medical/Surgical History** | **Medications** | **Allergies** | **Family History** |
|  | None | None | None |
| **Physical Examination** (Primary survey) (Secondary survey) | | | |
| **General** | Unresponsive. | | |
| **HEENT** | Patent airway, easily bag-mask ventilated (BMV) | | |
| **Neck** | Supple | | |
| **Lungs** | Shallow, course equal breath sounds, no stridor | | |
| **Cardiovascular** | Pale, mottled, white lips, no palpable pulses, Capillary refill (CR) = 10 seconds, cool skin | | |
| **Abdomen** | Abdomen soft, abrasions on arms and chest, no signs of head trauma | | |
| **Neurological** | Pupils 3 mm &minimally responsive, not responsive to painful stimuli, low tone, unresponsive, GCS 3 | | |
| **Skin** | No rash or bruises, mottled hands and feet | | |
| **GU** | Normal GU exam | | |
| **Psychiatric** | Unable to assess | | |

| Instructor Notes - Changes and CASE Branch Points | | |
| --- | --- | --- |
| **Intervention / Time point** | **Change in Case** | **Additional Information** |
| *Patient is rolled into ED resuscitation room.* | *HR 34 SpO2 85% BP Unable to obtain (UTO) RR BMV Temp 32deg C (rectal)*  *Patient dressed in wet clothes*  *Not crying*  *Shallow, course equal breath sounds with bagging*  *Sinus bradycardia, no murmur, 1+ pulses, CR=4-5 seconds* |  |
| *Learners establish team roles.* |  |  |
| *Assess airway, breath sounds, RR.*  *Establish need to intubate via rapid sequence intubation (RSI).*  *Assess adequacy of ventilation.*  *Monitors are applied to patient, including capnography end-tidal CO2(ETCO2) if available.*  *Place a C-collar.* | *ETCO2 monitoring: waveform with CO2 at 55 mmHg, other vital signs (VS) are unchanged.*  *Continuous ECG monitoring: sinus bradycardia* | *If bagging effectively, increase Oxygen saturation (SpO2) to 92%.* |
| *Assess circulation and continue CPR, initiating Pediatric Advanced Life Support (PALS) bradycardia algorithm with CPR with code dose epinephrine every 3-5min.*  *Assess adequacy of CPR with pulse checks.* |  | *RN attempts to obtain BP 2-3 times and is unable to do so. RN alerts the provider: “Doctor, we are unable to obtain a BP!” RN checks temperature and states, “Doctor, the temperature is 32 deg C!”* |
| *Recognize hypothermia, remove wet clothing, initiate rewarming with warmed intravenous fluid (IVF) or Baer hugger.*  *Take SAMPLE history.* |  | *Parent states: “What is happening to my child?!”*  *-Team member to explain what is occurring (interventions, patient status) in laymen’s terms, e.g. “We are trying to help him breath, help his heart work better with CPR, and help him get warmer.”* |
| *IV access is attempted and failed x 2 or attempted x 90 seconds (per PALS guidelines), IO access obtained, labs only attainable with IO.*  *Participant requests iSTAT labs: glucose, CBG, electrolytes.*  *Give 20cc/kg IVF bolus.* | *Glucose level is 100.*  *Capillary blood gas (CBG): 7.21/50/100/-14*  *Electrolytes: 138/4-100/22-14/0.5* | *If the labs are asked for, defer until IO is placed.*  *Parent asks questions:*   - *Is that hurting him?* - *Is he going to be OK?* - *What should I tell his dad?*   *-Team member to explain what is occurring (interventions, patient status) in laymen’s terms, e.g. “It might be painful, but is important that we perform CPR to make his heart work and that we place an IV/IO line to give him fluids and medicines to make him better. We are doing our best to make him comfortable.” “We are doing our best to help him.” “You can let his father know that we are trying to make him better by giving him medicine, performing CPR, and re-warming him.”* |
| *Electrocardiogram (ECG) requested*  *CXR requested* | *ECG: sinus bradycardia* | *Chest Xray (CXR) is en route.* |
| *Code dose epinephrine (0.01mg/kg of 1:10,000) given x 1* |  |  |
| **5 MINUTES INTO THE CASE** |  |  |
| *Reassess airway, breathing, circulation (ABCs).* | *Not crying*  *Coarse, crackly, equal breath sounds with ventilation,*  *Continuous ECG monitoring: ventricular fibrillation.*  *Pulses with compressions* |  |
| **BRANCHPOINT** |  |  |
| *Participants recognize ventricular fibrillation (Vfib) and initiate PALS cardiac arrest algorithm.*  *Defibrillate (~2J/kg).*  *Continue CPR for 2 minutes after 1^st^ defibrillation with ventilation.* | *If residents or newer trainees: After 1^st^ defibrillation, patient goes into normal sinus rhythm.*  *HR 55 SpO2 UTO BP UTO RR bagged T 34 degrees Celsius* |  |
| *If supervising physicians such as fellows, senior residents, or attending physicians:*  *Reassess patient.*  *Participants recognize persistent Vfib and continue PALs cardiac arrest algorithm.*  *Continue CPR for 2 minutes after 1^st^ defibrillation with ventilation.*  *Administer code dose epinephrine every 3-5minutes.*  *Defibrillate (~4J/kg).*  *Continue CPR for 2 minutes after 2^nd^ defibrillation with ventilation.* | *Continuous ECG monitoring: persistent ventricular fibrillation.*  *Not crying*  *Coarse, crackly, equal breath sounds with ventilation,*  *Pulses with compressions*  *After 2^nd^ defibrillation, patient goes into normal sinus rhythm.*  *HR 55 SpO2 UTO BP UTO RR bagged T 34 deg Celsius* |  |
| *Repeat assessment.* | *Non-responsive.*  *Airway patent, easily bag mask ventilated.*  *Breath sounds course, crackly bilaterally.*  *Pale, white lips, no palpable pulses, CR=6 seconds.*  *Pupils 3mm minimally responsive, low tone, unresponsive, GCS 3.*  *Abdomen soft, abrasions on arms and chest.* | *Parent asks questions:*   - *Is he OK – I see him moving?* - *Can you take that tube out of his mouth?* - *What are you shocking him?* - *Is the shock painful?*   *-Team member to explain what is occurring (interventions, patient status) in laymen’s terms, e.g. “We are doing our best to make sure his heart and circulation are improving.” “He needs that tube in his mouth to help him breathe.” “He needs to be shocked to help his heart improve.” “The shock can be painful if a patient is conscious, but he is not conscious and is unlikely to be feeling any pain right now. He needs the shock to help his heart work properly.”* |
| **10 MINUTES INTO THE CASE** |  |  |
| *Repeat assessment.* | *HR 88 (normal sinus rhythm) SpO2 93% BP 75/40 RR 16 T 36 degrees Celsius*  *Not crying, non responsive.*  *Intubate (or not), easily bagged with crackles throughout.*  *Regular rate and rhythm with 1+ pulses.*  *Pupils 3mm minimally responsive, not responsive to painful stimuli, low tone, unresponsive, GCS 3.*  *Abdomen soft, abrasions on arms and chest.* |  |
| *Stop chest compressions.*  *Continue ventilation.*  *Consider head computed tomography (CT).*  *Sign patient out to the Pediatric Intensive Care Unit (PICU).* |  | *Portable CXR arrives – provide with image as requested by team.* |

**Ideal Scenario Flow**

***Provide a detailed narrative description of the way this case should flow if participants perform in the ideal fashion.***

*The learners enter the room to find a patient being rolled in by EMS wearing only swimming trunks, who is apneic and unresponsive, with the medics actively bagging and performing chest compressions on the patient with an anxious mother en tow.*

*They immediately assign team role and place the patient on the bed in the resuscitation room. They place the patient on bedside monitors and recognize that the patient is hypoxic, bradycardic, hypotensive, hypothermic, and unresponsive. Effective bag mask ventilation and chest compressions are continued.*

*Team successfully intubates patient and continues to bag mask ventilate effectively. First ECG reveals sinus bradycardia. IV access fails x 3, team secures IO and obtain initial labs, start IVF 20cc/kg bolus, and order code dose epinephrine (0.01mg/kg of 1:10,000). Simultaneously, the team secures a cervical collar and begins to actively warm patient by removing swimming trunks, using warmed IVF and/or the BAER hugger.*

*After administering the first dose of epinephrine and starting warming, patient goes into ventricular fibrillation, recognizable on continuous ECG monitor. This is recognized immediately and patient is defibrillated with 2J/kg, with continued CPR for 2 minutes following defibrillation.*

*Repeat assessment reveals 1+ pulses, rising heart rate to >60 bpm, with sinus rhythm on the cardiac monitor, blood pressure measured at 75/40, SpO2 of 93% and the temperature rises to >34. The patient now has a secured airway and vascular access, is in sinus rhythm, is no longer hypoxic and is normothermic. Labs are notable for a metabolic acidosis. CXR notable for diffuse hazy opacities bilaterally. Head CT is pending. The providers continue to bag through the ET tube, monitor the vitals, reassess the A, B, C’s, and update the mother while they call the PICU for hospital admission.*

**Anticipated Management Mistakes**

***Provide a list of management errors or difficulties that are commonly encountered when using this simulation case.***

***For example:***

***ECG***

1. *Failure to recognize the need to effectively ventilate/establish a definitive airway. Most of our learners were able to recognize the need to ventilate using bag-mask ventilation, however, not everyone intubated during the scenario. We found reviewing this during our debriefing or advising a confederate to suggest this was helpful.*
2. *Failure to recognize the need for CPR. As CPR is ongoing on presentation, every learner continued CPR initially. Some learners did not resume CPR after interventions such as defibrillation or IV epinephrine or with rhythm change, leading to a delay in continued therapy. We found it helpful to have a confederate suggest these during the scenario if learners were struggling. In addition, you can turn up the volume on monitors, stop manikin pulses, or decrease pulse oximetry saturation as clues.*
3. *Failure to establish IO access after 3 failed IV access attempts. Most of our learners asked for an IO after PIV access failed x 2, however, if learners do not , consider having a confederate suggest this.*
4. *Failure to recognize bradycardia and follow PALS algorithm. All of our learners picked up the need to continue CPR in a bradycardic hemodynamically unstable patient. It may be helpful to have PALS cards available as well as continually decreasing the heartrate until the need for CPR is recognized.*
5. *Failure to recognize ventricular fibrillation and defibrillate. Most of our learners were able to recognize a shockable rhythm, however, were not always able to identify the rhythm as ventricular fibrillation. If not immediately recognized, turning up the volume on simulation monitors, having the rest of the patient’s vital signs decompensate, and finally, having a confederate suggest an intervention of defibrillation may be helpful. Having the person assigned to the defibrillator ask why a shock is indicated may prompt learners to verbalize the exact rhythm.*
6. *Failure to recognize hypothermia and actively re-warm. Most learners did not realize that the patient was hypothermic. Having the bedside RN state the temperature loudly is helpful. In one scenario, the confederate playing the patient’s mother stated “my baby looks so cold!” which prompted removal of the patient’s cold, damp clothes, and initiation of re-warming.*
7. *Dosing error and failure to adhere to dosing references. We had a Broselow tape and PALS cards available for learners to utilize as references.*
8. *Failure to consider trauma and secure c-spine precautions. Many learners did not consider trauma in this scenario and it might be helpful to have a confederate state concern about trauma or have a confederate parent state “I’m worried he hit his head!” to prompt learners to consider trauma. This should be discussed in the debrief.*
