# Supplementary material for: Pediatric Emergency Medicine Simulation Curriculum: Submersion Injury With Hypothermia and Ventricular Fibrillation
Source: MedEdPORTAL. 2017 Oct 17;13:10643. doi: 10.15766/mep_2374-8265.10643 (PMC6338133; doi:10.15766/mep_2374-8265.10643)
Supplement: Supplementary file 1 — A. Simulation Case.docx B. Environment Preparation.docx C. CXR ECG Rhythm Strip.docx D. Teamwork and Communication Glossary.docx E. Debriefing Materials.docx F. Session Evaluation Form.docx G. PowerPoint Presentation.ppt [file mep-13-10643-s001.zip › B. Environment Preparation.docx]

**Appendix B: Submersion Injury Simulation Environment Preparation**

Before each simulation, ensure the anticipated resuscitation equipment is available for the team’s use.

**Resources**

PALS reference cards, material

Patient Weight Estimator (e.g. Broselow Tape)

Pediatric Resuscitation Medication references (e.g.: Broselow tape, reference cards)

Documentation forms

**Universal Precautions**

Staff gowns

Gloves

Mask and face shields

**Medications (consider having all or only a limited number of medications available)**

| **Antibiotics:** | **Dose concentrations** |
| --- | --- |
| Ceftriaxone | 50-75 mg/kg |
| Vancomycin | 15 mg/kg |
|  |  |
| **Antipyretics:** | **Dose concentrations** |
| Acetaminophen | 15 mg/kg PO |
| Ibuprofen | 10 mg/kg PO |
|  |  |
| **Fluids:** | **Dose concentrations** |
| Normal Saline (warmed or room temperature) | 20 mL/kg IV for standard bolus |
| Hypertonic Saline (3%) | 3-5 mL/kg IV for hyponatremia-induced seizures |
| Lactated Ringers (warmed or room temperature) | 20 mL/kg IV for standard bolus |
|  |  |
| **Anti-epileptics:** | **Dose concentrations** |
| Fosphenytoin | 20 mg/kg IV for standard loading dose |
| Levetiracetam | 20 mg/kg IV for standard loading dose |
| Lorazepam | 0.1 mg/kg IV for status epilepticus |
| Midazolam | 0.2 mg/kg |
| Phenobarbital | 15-20 mg/kg IV for standard loading dose |
|  |  |
| **Resuscitation and RSI:** | **Dose concentrations** |
| Atropine | 0.02 mg/kg IV |
| Epinephrine | 0.01 mg/kg IV |
| Etomidate | 0.2 to 0.4 mg/kg IV |
| Fentanyl | 2-4 mcg/kg IV |
| Ketamine | 0.5-2 mg/kg IV |
| Propofol | 1.5-3 mg/kg IV |
| Rocuronium | 1 mg/kg IV |
| Succinylcholine | 1-2 mg/kg IV |

**Equipment**

Simulator in wet clothing (if not detrimental to simulator technology, clothing should be damp), on bed with patient identification band

Monitor – NIBP, HR, RR, Oxygen saturation, temperature and ETCO2 monitor (if available)

Blood Pressure cuff, Heart Rate monitor leads, Oxygen saturation probe, defibrillator cables and ETCO2 cannula (if available)

Oxygen hook-up on wall or cylinder

Bag-mask system, multiple size masks

O2 – nasal canula, mask - simple and/or non-rebreather

Suction

Thermometer, temperature probe

Nasal, oral airways, multiple sizes

Shoulder roll

Endotracheal tubes- 3.0, 3.5, 4.0, 4.5, 5.0, cuffed or uncuffed, stylets

Laryngoscope, Miller and Mac blades, multiple sizes

End-tidal CO2 colorimeter

Nasogastric tube(s)

Stethoscopes

IV/Angiocath, various sizes

IO needles, 2 sizes

Gauze, Tape

IV tubing/blood product tubing and filters

IV pumps, pressure bags/ blood product pumps

Syringes, multiple sizes

Bedside blood sample processors: glucose, electrolytes, gases

Specimen tubes

Crash cart & backboard

Defibrillator / AED

High flow nasal cannula system and/or nasal CPAP system
