# Supplementary material for: Pediatric Emergency Medicine Simulation Curriculum: Submersion Injury With Hypothermia and Ventricular Fibrillation
Source: MedEdPORTAL. 2017 Oct 17;13:10643. doi: 10.15766/mep_2374-8265.10643 (PMC6338133; doi:10.15766/mep_2374-8265.10643)
Supplement: Supplementary file 1 — A. Simulation Case.docx B. Environment Preparation.docx C. CXR ECG Rhythm Strip.docx D. Teamwork and Communication Glossary.docx E. Debriefing Materials.docx F. Session Evaluation Form.docx G. PowerPoint Presentation.ppt [file mep-13-10643-s001.zip › C. CXR ECG Rhythm Strip.docx]

Submersion Injury Chest Xray (CXR)


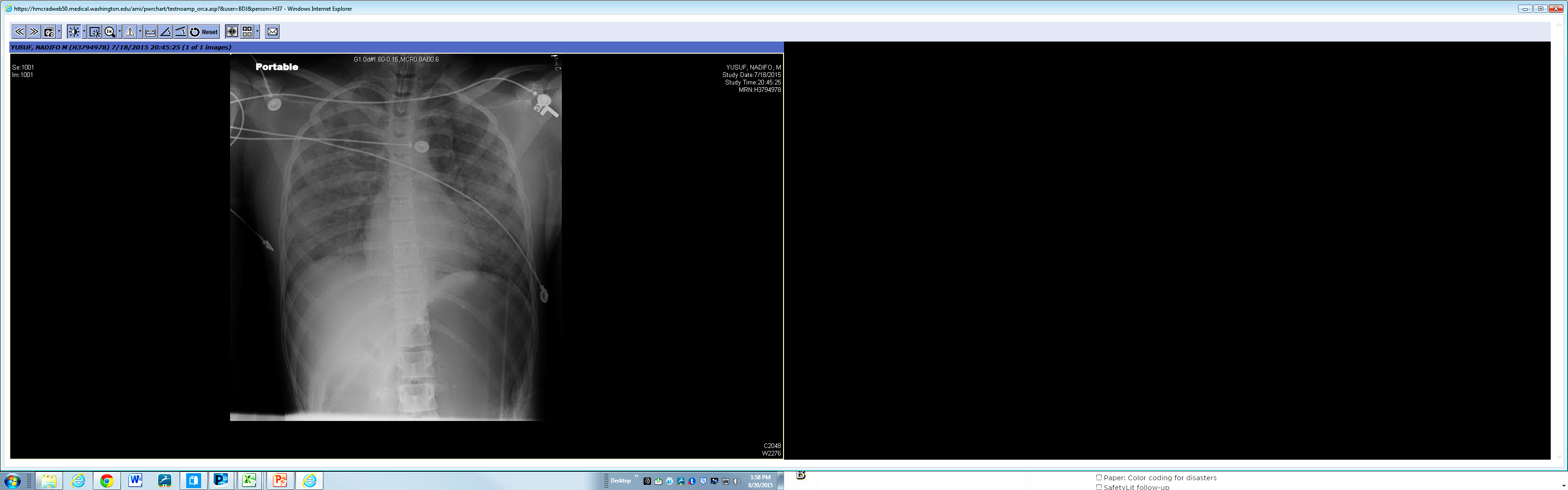


Courtesy of Brian Johnston, MD, MPH, Chief of Pediatrics Harborview Medical Center

Bradycardia ECG
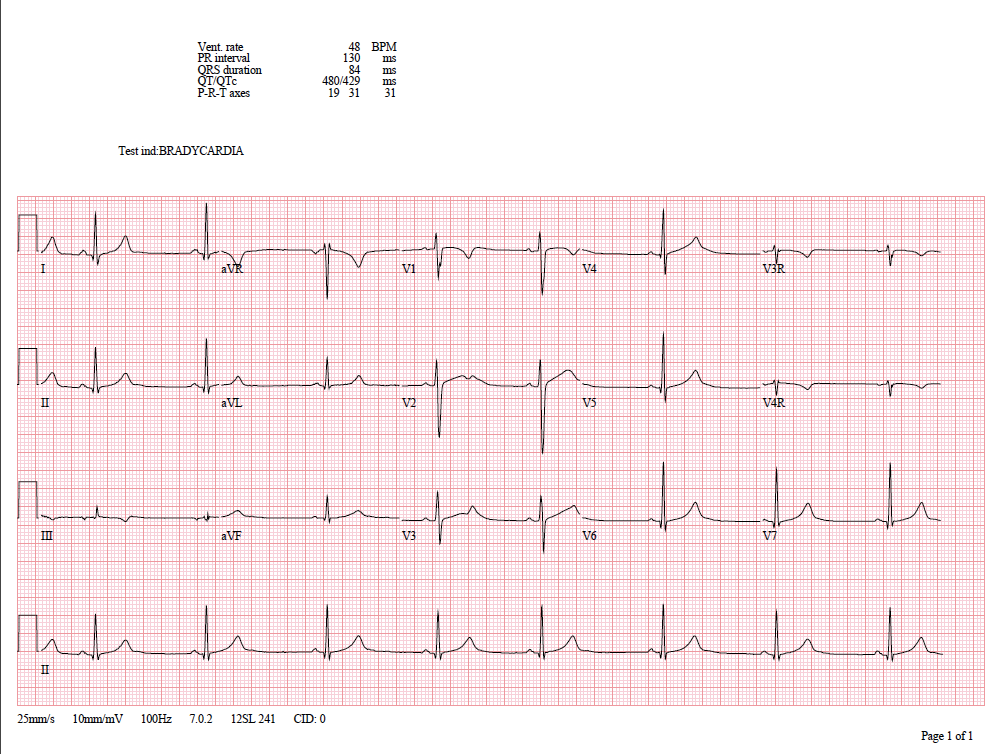


Courtesy of Derya Caglar, MD, Associate Professor of Pediatrics, Seattle Children’s Hospital

Ventricular Fibrillation Rhythm Strip


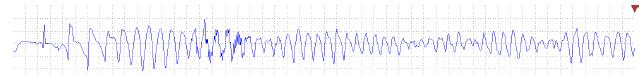


Courtesy of Troy Johnston, MD, Cardiology Program Director, Assistant Professor of Pediatrics, Seattle Children’s Hospital
