# Supplementary material for: Pediatric Emergency Medicine Simulation Curriculum: Submersion Injury With Hypothermia and Ventricular Fibrillation
Source: MedEdPORTAL. 2017 Oct 17;13:10643. doi: 10.15766/mep_2374-8265.10643 (PMC6338133; doi:10.15766/mep_2374-8265.10643)
Supplement: Supplementary file 1 — A. Simulation Case.docx B. Environment Preparation.docx C. CXR ECG Rhythm Strip.docx D. Teamwork and Communication Glossary.docx E. Debriefing Materials.docx F. Session Evaluation Form.docx G. PowerPoint Presentation.ppt [file mep-13-10643-s001.zip › E. Debriefing Materials.docx]

**Debriefing Overview**

*Simulation creates the opportunity to debrief. We believe that the focus of each simulation should be the DEBRIEF. Simulation creates the opportunity to examine our medical management, technical skills and teamwork and communication skills. It facilitates discussion about challenges in a safe environment in order to improve the quality of patient care.*

**Framework for debriefing:**

Each debrief should consist of four components:

 Introduction

 Discussion of emotions

 Discussion of medical management and technical skills

 Discussion of teamwork and communication skills

*There is often overlap between medical management and teamwork issues. Debriefing may not follow a linear progression of all four of these components.*

General Debriefing Goals:

 Try to facilitate the **team’s** discussion (avoid dominating the conversation)

 Ask open-ended questions (avoid yes/no questions)

 Discuss the team performance (not the individual)

**1) Introduction**

This “sets the stage” for debriefing and creates expectations.

What you might say:

 This is an opportunity to reflect and learn, improve our medical care, teamwork, and communication.

 Everyone should be able to ask questions and share their thoughts.

 Once you leave this session, we encourage open discussion of the concepts, but ask you to not to

discuss individual performance.

 Remind the group of the ground rules (treat everyone with respect, maintain confidentiality).

**2) Emotional experience discussion**

There are a couple of camps regarding discussing emotions in debriefing simulation scenarios. One perspective is that until emotions are dealt with, it can difficult for adult learners to “move on”: switching gears to process thoughts, actions and opportunities for improvement. Another perspective is that adult learners should process their emotions independently.

Our perspective is the first based on our experience with various simulation scenario debriefings at our institution. If a group or team member is emotionally charged (sad, mad or frustrated) regarding something that did or did not happen in a scenario, it is usually difficult for the individual or the group to be actively engaged, receptive to feedback and able to promote learning, until the emotions are addressed.

An example: a medication error occurs. One team member may think it is all his/her fault. S/he may feel embarrassed, judged, etc. If he/she can verbalize this, other team members may offer different perspectives, which enable the team to process the error together, potentially identifying contributing systems issues. If the emotions are not addressed- team members may feel embarrassed, responsible and not engage in a discussion, failing to identify systems issues which led to the error.

What you might say:

 How did that feel?

 Can you tell me more? Why?

**3) Medical management and technical skills**

This portion of the discussion focuses on the medical aspects of the scenario. It’s usually more comfortable to begin with these “facts.”

What you might say:

 Let’s begin by discussing medical management.

 What did you think was wrong with the patient? Can someone briefly summarize what happened in this scenario?

 How did you reach those conclusions?

**4) Teamwork and communication (a. k. a. crew resource management, non-technical skills)**

This portion of the discussion focuses on how the team worked together. It can be emotionally charged and difficult to discuss without feeling personal. The challenge is to try to generalize specifics into themes.

What you might say:

 Let’s talk about how you functioned as a team.

 What did your team do well?

 What could your team do differently next time?

 That is something I see often. Has anyone else experienced that? How have you seen that handled?

**5) Summarizing**

 This is your opportunity to ensure the key learning points are highlighted.

 Try to identify approximately three take-home points.

 You may ask the participants’ to identify take home points or call them out yourself.

*Medical management/technical skills examples:*

1. This was a scenario of pediatric submersion injury.
2. Focusing on the primary survey and having a systematic work up are critical for effective resuscitation. Evaluation and management of submersion injury: history, physical examination, IV/IO access and resuscitation, ventilation and intubation if needed to protect airway, management of hypothermia and cardiac sequelae of bradycardia and ventricular fibrillation, recognition of potential for trauma.

*Teamwork/communication examples:*

(c) Recognize need for a full resuscitation team when a patient presents with likely submersion injury.

(d) Designate leadership and team member roles to ensure coordinated team functioning.

(e) Use *brief* or *huddle* to create a shared mental model for the working diagnosis and management plan.

Below are examples of learning objective based statements & questions you may use to debrief the team.

| **Examples of debriefing for different learning objectives** | | |
| --- | --- | --- |
| **Recognizing submersion injury** | | |
| Debriefer Script | Reference Material | Instructor Notes |
| I noticed you *(were quick/took a while)* to include submersion injury with hypothermia and cardiac arrhythmia on the differential diagnosis. This was (*great/could lead to delays)* since delays in recognition can result in clinical deterioration.   What were your thought processes around what was occurring?   What helped/hindered you in deciding the A&P? | Risks/signs of submersion injury:   Patient age, witnessed event, history, preceding symptoms   Drowning, respiratory arrest, GCS 3, hypothermia, bradycardia followed by ventricular fibrillation |  |
| **Initial identification and management of submersion injury** | | |
| Debriefer Script | Reference Material | Instructor Notes |
| I noticed you (*were quick/ could have been quicker*) to recognize hypothermia and/or bradycardia and/or ventricular fibrillation. This was (*great/could lead to delays)* in clinical stabilization.   How did your team decide on the management priorities?   What helped/hindered you?  ***OR***  I noticed you *(were complete/missed some opportunities)* in initially evaluating for the signs and symptoms of submersion injury. This was *(great/could have been even better)* because early identification and management could lead to improved outcomes*.*   How did your team decide on the evaluation priorities?   What helped/hindered you? | Initial management of submersion injury:   Assess airway, breathing circulation   Ventilation   CPR   Obtain access (IV/IO)   Epinephrine for bradycardia   Assess mental status   Preform secondary exam and gather history   Defibrillate for Vfib (once occurs later in scenario)   Send imaging and laboratory studies |  |
|  |  |  |
|  |  |  |

| **Examples for debriefing different Teamwork Learning Objectives** | | |
| --- | --- | --- |
| **Roles and Responsibilities** | | |
| Debriefer Script | Reference Material | Instructor Notes |
|  Let’s talk about how you functioned as a team.   From my perspective it looked like you (*did/did not) have* a clear team leader and defined team roles. I think this is (*great/concerning)* because clear team roles can help a team function smoothly- improving how quickly interventions take place and reducing errors.  o How did you function as a team?  o What did you think about your roles? | Team leader  ▪ Clear direction, coordination, timely interventions  ▪ Foot of patient  ▪ Check-back communication for med dosing  ▪ Use dosing reference (Appendix B)  Airway/Procedure MD  ▪ Manage airway  ▪ Head of patient  Survey MD  ▪ Primary, Secondary survey, pulses with CPR, reassess  Nursing roles  ▪ Medication Prep (draw-up meds)  ▪ Medication Admin (give meds)  ▪ Documenting (time keeper)  ▪ Check-back communication for med dosing  ▪ Use dosing reference (Appendix B) |  |

| **Brief and Huddle** | | |
| --- | --- | --- |
| Debriefer Script | Reference Material | Instructor Notes |
| I noticed that your team *(did/didn’t/took a while to)* (*brief prior to the initial patient assessment/huddle after the initial evaluation).* I thought this was (*great/could have helped you work better as a team*) in order to facilitate patient care.   What *(helped/hindered)* your team from (*briefing/huddling*)?   How did that impact your team?   What could your team have done differently?   How can you make sure that  *(does/doesn’t*) happen again? | The goal of a brief/huddle is to create a shared mental model. Assure all team members know what the working diagnosis is, management priorities and next steps in care.  ▪ Everyone on the team is responsible for making this happen. Anyone can ask for a brief/huddle. Brief/huddle is usually led by team leader.  ▪ If one team member doesn’t know what’s up or what’s next- s/he is probably not alone. |  |

| **Directed call out** | | |
| --- | --- | --- |
| Debriefer Script | Reference Material | Instructor Notes |
| I noticed that you (*did/didn’t/intermittently*) used (*peoples names/roles/eye contact*) when (*calling out orders/asking for assistance*). I thought this was (*great/could have been more directed*) in order to facilitate communication.   What did you notice about orders/questions that were asked?   How did this impact your team? | Directed call out. A tactical communication skill to assure that important orders/questions are specifically directed to one individual (rather than called out into the air).  Example:  ▪ “Jennifer-What’s the SaO2%?”  ▪ “Kim- Give normal saline 500 mL.”  ▪ “Team leader- she stopped responding to pain.” |  |

| **Closed loop communication/Check back** | | |
| --- | --- | --- |
| Debriefer Script | Reference Material | Instructor Notes |
| I noticed that you used closed-loop communication *(consistently/a lot/rarely)*. Closed-loop communication can be critical for catching errors and assuring that *(information/an order/a request)* is heard.   How were the communication loops in the team?   How did that impact your team?   Has anyone seen problems with this in a patient resuscitation?   Has anyone seen closed loop communication prevent an error?   How could you do it differently next time? | Closed loop communication/check back is a strategy that requires verification of information. This enables the sender of the message to verify it has been heard and heard correctly. It enables the receiver to confirm what they heard is correct.  ▪ Team leader “Call for EKG”  ▪ Float nurse “calling technician for an  EKG”  ▪ Team leader “correct” |  |

**Submersion Injury** **Medical** **Management** **Evaluation/Debriefing** **Form**

This checklist identifies core medical management /technical skills. It’s hard to discuss more than 3 of these during one debriefing session. We recommend focusing on 2-3 of these issues.

**Assessment of ABCDE’s □** Done Well **□** Needs Work

Specific comments:

*Discussion Points: What did you think of the assessment of the ABCDE’s? What could you do differently?*

**Avoiding premature diagnostic closure □** Done Well **□** Needs Work

Specific comments:

*Discussion Points: What other potential etiologies could cause these signs and symptoms? Differential diagnoses may include but are not limited to toxic exposure, trauma, primary arrhythmia, seizure, non accidental submersion. How did you decide* *what the most likely cause was? What other studies and therapies would you pursue in the case?*

**Recognizing submersion injury □** Done Well **□** Needs Work

Specific comments:

*Discuss Points: What are signs of submersion injury? Witnessed submersion, difficulty breathing, excessive coughing. .*

**Initial management of submersion injury □** Done Well **□** Needs Work

Specific comments:

*Discuss Points: What’s the emergent management for submersion injury? Management of airway, breathing (need for supplemental oxygen or definitive airway) and circulation (assessment of arrhythmias),exposure (temperature assessment and rewarming), disability.*

**Submersion Injury** **Teamwork** **and** **Communication** **Evaluation**

This checklist identifies core teamwork and communication skills. It’s hard to discuss more than 3 of these during

one debriefing session. We recommend focusing on 2-4 of these issues.

**Leader/Roles Identified & Maintained □** Done Well **□** Needs Work

Specific comments:

*Discussion Points: What helped/hindered having clear leadership and roles?*

**Directed Call out □** Done Well **□** Needs Work

Specific comments:

*Discussion Points: How were orders given- “Into the air” or directed at specific individuals? How did that impact*

*you? How could they be delivered more effectively?*

**Check back/Closed loop communication □** Done Well **□** Needs Work

Specific comments:

*Discussion Points: Describe closed loop communication.*

**Shared Mental Model □** Done Well **□** Needs Work

Specific comments:

*Discussion Points: How did team members share information/working diagnosis/management plan*

*(brief/huddle)?*
