# Supplementary material for: Pediatric Emergency Medicine Simulation Curriculum: Submersion Injury With Hypothermia and Ventricular Fibrillation
Source: MedEdPORTAL. 2017 Oct 17;13:10643. doi: 10.15766/mep_2374-8265.10643 (PMC6338133; doi:10.15766/mep_2374-8265.10643)
Supplement: Supplementary file 1 — A. Simulation Case.docx B. Environment Preparation.docx C. CXR ECG Rhythm Strip.docx D. Teamwork and Communication Glossary.docx E. Debriefing Materials.docx F. Session Evaluation Form.docx G. PowerPoint Presentation.ppt [file mep-13-10643-s001.zip › F. Session Evaluation Form.docx]

**Appendix F: Submersion Injury Simulation Session Evaluation Form**

**Instructor:** ____________________________ **Date:**  _________________

**Case Presented:** Submersion Injury

|  | Strongly  Disagree | Disagree | Neutral | Agree | Strongly  Agree |
| --- | --- | --- | --- | --- | --- |
| 1. This simulation case provided is relevant to my work. | 1 | 2 | 3 | 4 | 5 |
| 1. The simulation case was realistic. | 1 | 2 | 3 | 4 | 5 |
| 1. This simulation case was effective in teaching basic resuscitation skills. | 1 | 2 | 3 | 4 | 5 |
| 1. I feel prepared to perform a primary survey on a patient with a submersion injury. | 1 | 2 | 3 | 4 | 5 |
| 1. This scenario prepared me to elicit critical history from a patient with a submersion injury. | 1 | 2 | 3 | 4 | 5 |
| 1. I feel comfortable activating team assistance early in a resuscitative event. | 1 | 2 | 3 | 4 | 5 |
| 1. This scenario allowed me to practice effective teamwork and communication skills. | 1 | 2 | 3 | 4 | 5 |
| 1. I can recognize the degree and progression of submersion injuries. | 1 | 2 | 3 | 4 | 5 |
| 1. This simulation case was effective in teaching submersion injury management skills. | 1 | 2 | 3 | 4 | 5 |
| 1. I feel equipped to discuss prognosis and prevention of submersion injuries with families. | 1 | 2 | 3 | 4 | 5 |
| 1. The debrief created a safe environment. | 1 | 2 | 3 | 4 | 5 |
| 1. The debrief promoted reflection and team discussion. | 1 | 2 | 3 | 4 | 5 |

Can you list/describe 1 or more ways this simulation session will change how you do your job

How can we improve this scenario?

Comments:
